# Genome-Wide Identification and Expression Analysis of PkNRT Gene Family in Korean Pine (*Pinus koraiensis*)

**DOI:** 10.3390/plants14020238

**Published:** 2025-01-16

**Authors:** Xinyu Zhao, Haibo Wu, Boyang Li, Pengyang Wang, Peng Zhang, Hailong Shen, Jianfei Yang

**Affiliations:** 1 College of Forestry, Northeast Forestry University, Harbin 150040, China; 18638613878@163.com (X.Z.); whb152407@163.com (H.W.); liboyang_666@163.com (B.L.); 15254465519@163.com (P.W.); zhangpeng@nefu.edu.cn (P.Z.); 2 Key Laboratory of Sustainable Management of Forest Ecosystem, Ministry of Education, Harbin 150040, China; 3 State Forestry and Grassland Administration Engineering Technology Research Center for Korean Pine, Harbin 150040, China

**Keywords:** Korean pine, NO_3_^−^ transporters, NO_3_^−^ deficiency, lateral root development

## Abstract

The utilization of nitrogen (N) is crucial for the optimal growth and development of plants. As the dominant form of nitrogen in temperate soil, nitrate (NO_3_^−^) is absorbed from the soil and redistributed to other organs through NO_3_^−^ transporters (NRTs). Therefore, exploration of the role of NRTs in response to various NO_3_^−^ conditions is crucial for improving N utilization efficiency (NUE). Here, we present a comprehensive genome-wide analysis and characterization of the NRT gene family in Korean pine, an invaluable tree species cultivated extensively in northeastern China. A total of 76 *PkNRTs* were identified in Korean pine and further divided into three subfamilies (NRT1/NPF, NRT2, and NRT3) based on phylogenetic analysis. All *PkNRTs* were distributed on 11 chromosomes, with multiple tandem duplications observed. The tissue-specific expression analysis indicated that most *PkNRTs* showed differential expression in six vegetative tissues. Furthermore, a significantly greater number of lateral roots was observed in seedlings under nitrogen-deficient conditions, accompanied by an increase in both total root biomass and root length. The temporal expression profiles of 16 *PkNRTs* in seedling roots revealed that four *PkNRTs*, *PkNPF5.6*, *PkNPF5.13*, *PkNPF6.1*, and *PkNPF6.2*, exhibited significantly upregulated expression under the NO_3_^−^ deficiency condition, whereas robust induction was observed for *PkNPF1.1*, *PkNRT2.6*, and *PkNRT3.3* upon the NO_3_^−^ sufficiency condition. The expression patterns of the *PkNRTs* suggest their potential diverse roles as key participants in root NO_3_^−^ uptake under varying NO_3_^−^ conditions during root development. These findings would provide a theoretical foundation for further investigations into the functions of *PkNRTs* in Korean pine.

## 1. Introduction

Nitrogen (N) is an essential macronutrient for most of higher-plant growth and productivity, playing critical roles in various physiological and metabolic processes [1,2]. The dominant form of N in temperate soils is nitrate (NO_3_^−^), accompanied by ammonium (NH_4_^+^), amino acids, and other N-containing substances. The appropriate application of N can enhance crop yields, whereas the excessive use of N may have opposite effects on plants [3,4]. Therefore, the understanding of regulatory mechanisms underlying NO_3_^−^ acquisition processes is crucial for optimizing plants’ N use efficiency (NUE) capacity.

Roots play a vital role in N absorption from the soil for plants. Plants adapt their N uptake and transport mechanisms through modification in root architecture and the optimization of metabolic processes [5]. The initial step in triggering nitrogen responses is activating N absorption mediated by transporters at the root surface. To adapt to different concentrations of NO_3_^−^ in soil, plant roots have evolved a low-affinity transport system (LATS, >1 mM) and high-affinity transport system (HATS, <1 mM) for assisting NO_3_^−^ uptake [6]. Most NO_3_^−^ obtained by plants from soil is actively transported through a group of NO_3_^−^ transporters (NRTs), which constitute a diverse family with many members and distinct functions [7,8]. Several genes from the NRT family have been identified in HATS and LATS with apparent function. Based on sequence homology and functional characteristics, NRTs are primarily grouped into three subfamilies: the Nitrate Transporter 1/Peptide Transporter (NRT1/PTR, also known as NPF), Nitrate Transporter 2 (NRT2), and NRT3 (as known as NAR) families [9,10]. NRT1 and NRT2 function as NO_3_^−^ transporters responsible for LATS and HATS, respectively [11]. Unlike NRT1 and NRT2, NRT3 members lack the inherent ability for NO_3_^−^ transportation; instead, they play important roles in high-affinity NO_3_^−^ transport by regulating the activity of NRT2 proteins [12]. In addition to their involvement in NO_3_^−^ transport, NRT proteins have been reported to participate in a diverse range of physiological processes in plants, including shoot/root development, hormone transportation, and stress signaling response [13,14].

Up to now, many members in the NRT family have been identified in higher plants, while only a few have been well characterized. Among the three subfamilies, the NRT1/NPF clade exhibits greater a abundance of members than the NRT2 and NRT3 subfamilies, indicating the significant roles of NRT1/NPF in NO_3_^−^ absorption, translocation, and assimilation [15,16]. *AtNRT1.1* (also known as CHL1/NPF6.3) was the first identified member of the NRT family in Arabidopsis, which has dual affinity (low and high affinity) for NO_3_^−^ absorption and root-to-shoot transport [17,18]. Except for *AtNRT1.1*, most NRT1 members exhibited low affinity. For example, *NPF4.6/NRT1.2* and *NPF2.7/NAXT1* had low affinity for NO_3_^−^ absorption, and were also shown to be involved in root NO_3_^−^ uptake, with *NPF4.6* acting on NO_3_^−^ influx and *NPF2.7* involved in NO_3_^−^ efflux, respectively [19,20]. The other identified NRTs have been reported to play a crucial role in the internal transport of NO_3_^−^, including processes such as xylem and phloem loading, as well as translocation to leaves or seeds [21]. The *OsNPF2.4* functions as a low-affinity transporter in the uptake of NO_3_^−^ and N recycling in rice [22]. The mutations in *AtNRT1.9/NPF2.9* resulted in a decrease in NO_3_^−^ content within the root xylem and an increase in NO_3_^−^ content within the shoot, thereby impacting the allocation of NO_3_^−^ between the root and shoot [23]. In Arabidopsis, HATS is mediated by the interaction between NRT2 with *NPF6.3*. The expression of *AtNRT2.4* is upregulated in both roots and shoots in response to N starvation. The overexpression of *AtNRT2.4* enhances the facilitation of NO_3_^−^ absorption by the roots and its subsequent translocation from the roots to the phloem [24]. Although the characterization of some NRTs has been accomplished, there remains a lack of comprehension regarding the functions of the majority of NRTs in N uptake, transport, and utilization. Therefore, further investigation and verification of NRT protein functions in plants are essential for advancements in N fertilization.

Korean pine (*Pinus koraiensis*) is an invaluable tree species in northeastern China, holding significant ecological, economic, and social importance [25,26]. Because of the high content of various nutrients in pine nuts and excellent wood properties, Korean pine is cultivated extensively in northeast China. The current limitation of forest growth in northern China is due to the low availability of nitrogen in the soil. Hence, N fertilization plays a vital role in expediting the growth rate of Korean pine, given its slow growth rate and lengthy reproductive cycle. Its NUE can be effectively improved by increasing the absorption capacity of NO_3_^−^ in roots and the redistribution capacity of NO_3_^−^ in other tissues [27]. Though great progress has been achieved for plant NRT genes, there is a lack of research on gymnosperms, particularly Korean pine.

In the present study, genome-wide identification and comprehensive bioinformatics analyses of the NRTs in Korean pine were performed. A total of 76 NRTs were identified and characterized, including chromosomal location, genetic structure, and the phylogenetic relationship of the PkNRT gene family. The tissue-specific expression pattern of the *PkNRTs* was also explored. Furthermore, the phenotypic responses of Korean pine seedlings to nitrogen deficiency treatments were evaluated, along with the expression profiles of specific *PkNRTs* expressed in roots. These findings will facilitate the identification of *PkNRT* functions, thereby offering new insights into elucidating the molecular regulatory network of nitrogen uptake and transport in Korean pine.

## 2. Results

### 2.1. Characterization of the PkNRT Gene Family Members in Korean Pine

A total of 76 PkNRT proteins were identified that contained NRT or NRT-like repeats, including 66 NRT1/NPF, 6 NRT2, and 4 NRT3 members, in Korean pine (Table 1). The predicted number of amino acids varies widely among the three subfamilies of *PkNRTs*. The number of amino acids in the NPF and NRT2 subfamilies ranges from 533 (*PkNRT2.4*) to 661 (*PkNPF2.1*), with the corresponding molecular weight ranges from 57.87 kDa to 73.07 kDa. In contrast, the members in the NRT3 subfamily have notably shorter amino acid sequences (207aa–268aa) and lower molecular weights (22.58–23.98 kDa). The theoretical predicted isoelectric point (pI) range of NRT proteins is 5.25 to 9.73, and the pI values of most members (65/76) are greater than 7, indicating that most NRT proteins tend to be positively charged at a physiological pH. In terms of the hydrophobicity analysis, most *PkNRTs* are hydrophobic, and only *PkNRT3.4* possesses the negative GRAVY value, indicating its potential as a hydrophilic protein. Additionally, the number of transmembrane domains (TMs) in the NPF and NRT2 proteins is between 8 and 12, except for the NRT3 protein, which has only 1 TM.

### 2.2. Phylogenetic Analysis of PkNRTs

**Figure 1:**
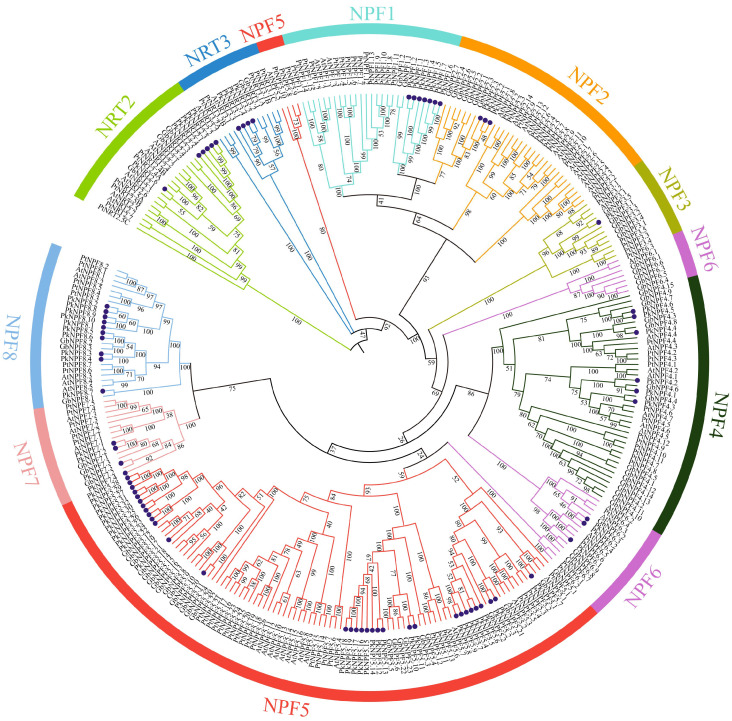
Phylogenetic analysis of NRT proteins from *Arabidopsis thaliana*, *Populus trichocarpa*, *Ginkgo biloba*, and *Pinus koraiensis.* The NRT proteins were classified into three subfamilies: NPF/NRT1, NRT2, and NRT3. NPF/NRT1 can be further classified into eight subfamilies (NPF1–NPF8). The dark blue dots indicate the PkNRT proteins.

The phylogenetic relationships among the NRT gene family members in Arabidopsis, Poplar, Ginkgo, and Korean pine were elucidated through the construction of a phylogenetic tree. As expected, the *PkNRTs* were divided into three subfamilies: NPF/NRT1, NRT2, and NRT3 (Figure 1). The NPF subfamily comprised the majority of the NPF/NRT1 members, and it was further classified into eight clusters (NPF1–NPF8). Among them, NPF5 has the most members (*NPF5.1*–*NPF5.32*), and NPF3 has the fewest member (*NPF3.1*). The members of NPF5 were further categorized into three distinct subgroups. The PkNPF1 and PkNPF2 subfamilies are closely positioned on the phylogenetic tree, sharing the same branch, which suggests that these subfamilies may be evolutionarily closely related. In addition, the *PkNRTs* were found to cluster more closely with those of Ginkgo than those of Arabidopsis, indicating that Korean pine and Ginkgo may be more closely related evolutionarily.

### 2.3. Motifs, Conserved Domain, and Gene Structure Analysis of PkNRTs

**Figure 2:**
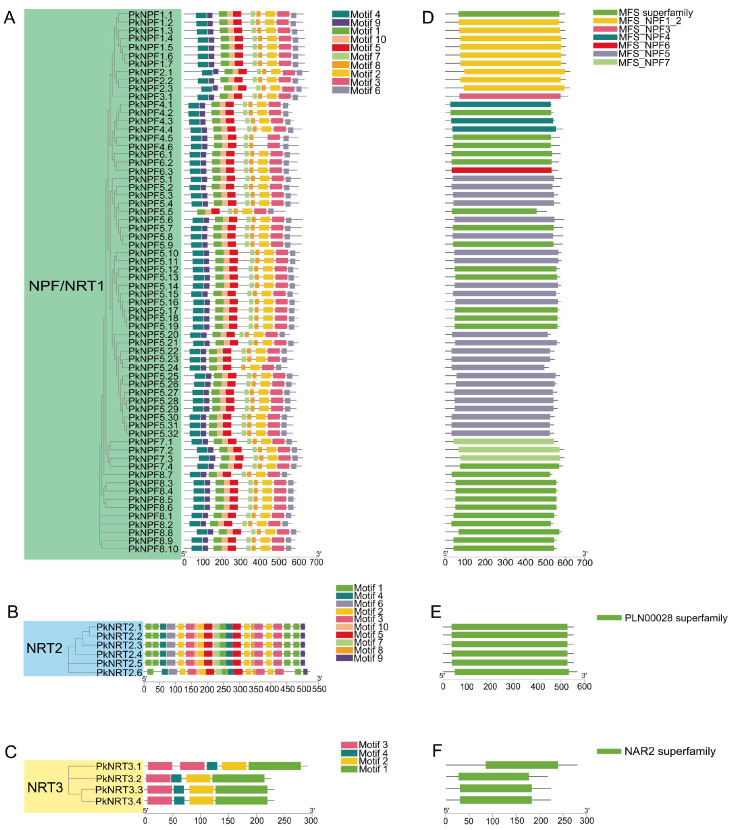
The motif, gene structure, and conserved domains of *PkNRTs*. (**A**–**C**) The motif analysis of NRT1/NPF, NRT2, and NRT3 proteins. (**D**–**F**) The conserved domains of *PkNRTs*. All detailed information of identified conserved motif sequences is provided in Table S1. Gene structure detailed information is provided in Table S2. A scale bar is provided at the bottom.

After analyzing the conserved motifs, it was observed that a majority of *PkNRTs* exhibited a consistent distribution pattern and contained all identified motifs (Figure 2A–C). Only a few genes displayed atypical motif distributions, such as *PkNPF5.5* in the NPF subfamily, *PkNRT2.6* in the NRT2 subfamily, and *PkNRT3.1* in the NRT3 subfamily. Although the motifs in the NPF subfamily are highly conserved, gene structure analysis revealed that certain genes within this subfamily, such as *PkNPF4.5*, *PkNPF4.6*, *PkNPF5.6*, *PkNPF8.9*, *PkNPF8.10*, *PkNPF8.1*, and *PkNPF8.8*, possess much longer introns, which impact the length of their genomic sequences (Table S2). Through the analysis of conserved structural domains, it was found that the NPF subfamily has different superfamily structural domains, including MFS_NPF1_2(cd17416), MFS_NPF3(cd17415), MFS_NPF4(cd17414), MFS_NPF5(cd17417), MFS_NPF6(cd17413), MFS_NPF7(cd17419), and the MFS superfamily(cl28910) (Figure 2D). The NRT2 and NRT3 subfamilies have only one highly conserved structural domain, named the PLN00028(cl30556) and NAR2(cl25236) domains, respectively (Figure 2E,F).

### 2.4. Analysis of Chromosomal Distribution and Collinearity of PkNRTs

**Figure 3:**
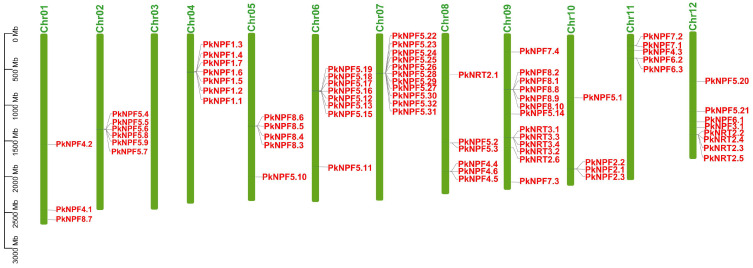
Chromosomal location of *PkNRTs*. The chromosome numbers are shown above. The unit of scale is in megabases (Mb).

According to the chromosomal location analysis, it was found that the *PkNRTs* were distributed on 11 chromosomes of Korean pine, except for Chr03 (Figure 3). The density of *PkNRTs* also exhibited variation among the chromosomes. Chr09 possessed the highest number of *PkNRT* genes, whereas Chr01 had only three *PkNRTs*. For the NPF subfamily members, most members of PkNPF5 were found on Chr02, Chr06, and Chr07, which aligns with the three subgroups identified in the phylogenetic analysis. The distribution of PkNPF8 proteins was observed on Chr05 and Chr09, whereas all members of PkNPF1 and PkNPF2 were found to be located on Chr04 and Chr10, respectively. The members of the NRT3 subfamily, similarly to those of PkNPF1 and PkNPF2, exhibited exclusive distribution on Chr09. Additionally, for the NRT2 subfamily, four out of the six NRT2 members were distributed on Chr12. It is worth noting that a large number of *PkNRTs* were clustered in adjacent positions on the chromosome (such as the PkNPF2, PkNPF5, PkNPF8, PkNRT2, and PkNRT3 subfamilies), indicating that the *PkNRTs* may have undergone frequent duplication events during evolution.

**Figure 4:**
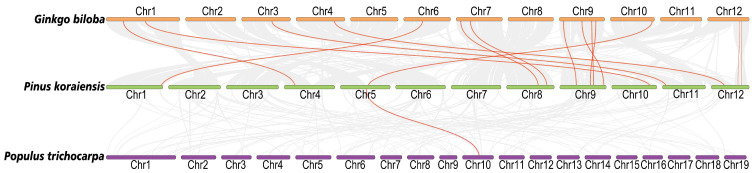
Collinearity analysis of NRTs between *Ginkgo biloba* and *P. koraiensis* and *P. trichocarpa*. The gray lines represent the collinearity between *P. koraiensis* and other two species. Collinear gene pairs in the NRT gene family are marked with red lines.

The analysis of collinearity between species is of great significance for further studying the evolutionary process of the NRT family. Through collinearity analysis between Korean pine, Poplar and Ginkgo, it was found that Korean pine had 14 direct collinear gene pairs with Ginkgo, and these genes were included in the PkNPF and PkNRT2 subfamilies (Figure 4, Table S3). In contrast, the evolutionary relationship between Korean pine and Poplar was relatively distant, with only one collinear gene pair belonging to the NPF8 subfamily in common. Furthermore, we conducted a collinearity analysis comparing Korean pine with rice and Arabidopsis, two model plants in angiosperms. The results revealed no presence of a collinear gene pair in *NRTs* among Korean pine, rice, and Arabidopsis (Figure S1). In addition, the ratio of non-synonymous (Ka) to synonymous substitution (Ks) rates was calculated for the 15 direct collinear gene pairs to determine whether there was selective pressure acting on these protein-coding genes (Table S3). The Ka/Ks ratios of the NRT gene pairs in these species are less than 1, indicating that these genes primarily underwent purifying selection during evolution. This purifying selection suggested that deleterious non-synonymous mutations are selectively eliminated to preserve their function, thereby maintaining their crucial roles in NO_3_^−^ absorption and transport. In addition, we calculated the Ka/Ks ratios of tandemly duplicated gene pairs in *PkNRTs* (Table S4). The results revealed that most gene pairs have undergone purifying selection (Ka/Ks < 1), indicating that these *NRT* genes were relatively conserved during the evolutionary process.

### 2.5. Characterization of CREs in the Promoter Regions of PkNRTs

To further investigate the potential functions of the NRT gene family, the cis-regulatory elements (*CREs*) were analyzed (Figure S2). The results showed that *CREs* identified in the promoter of *PkNRT* genes were mainly divided into three categories: plant development, hormones, and stress (Table S5). Among them, *CREs* related to plant development were CCAAT-box, which plays an important role in early plant growth and tissue differentiation. The *CREs* associated with abiotic stress responses, such as the MBS element implicated in drought induction and the LTR element involved in cold response, were also found in *PkNRT* promoters. Additionally, the hormone-responsive elements were enriched in *PkNRT* promoters (Figure S2), including auxin response elements (AuxRR-core, TGA-box, and TGA-element), gibberellin response elements (GARE-motif and P-box), abscisic acid response elements (ABRE), salicylic acid response elements (SARE and TCA-element), and jasmonic acid response elements (CGTCA-motif and TGACG-motif), which suggests that hormones may play a potential role in regulating the expression of *PkNRTs*.

### 2.6. Transcript Abundance Analysis for Different Tissues in PkNRTs

**Figure 5:**
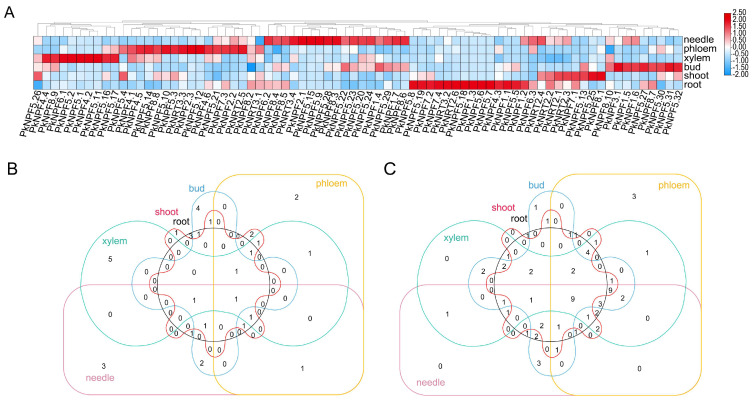
Transcriptome-based expression profiling of *PkNRTs*. (**A**) Expression of *PkNRTs* from six Korean pine tissues including root, shoot, bud, xylem, phloem, and needle. (**B**) The Venn diagram of the top 12 most highly expressed *PkNRTs* involved in each tissue. (**C**) The Venn diagram of *PkNRTs* with the lowest expression levels (FPKM < 0.5) between different tissues.

To explore the tissue-specific expression of *PkNRTs*, we conducted further investigation into their expression pattern by utilizing transcriptome data obtained from six different tissues (needle, phloem, xylem, bud, shoot, and root) (Table S6). The expression patterns of certain genes in specific tissues were observed (Figure 5A). For example, *PkNPF1.1*, *PkNPF1.2*, *PkNPF1.3*, *PkNPF4.3*, *PkNPF5.6*, *PkNPF5.7*, *PkNPF5.18*, and *PkNRT3.2* exhibited high expression levels specifically in the root. A total of 13 NRTs were highly expressed in the needle, including *PkNPF1.5*, *PkNPF2.1*, *PkNPF2.2*, *PkNPF3.1*, *PkNPF5.2*, *PkNPF5.9*, *PkNPF5.15*, *PkNPF5.21*, *PkNPF6.2*, *PkNPF7.3*, *PkNPF8.5*, *PkNRT2.5*, and *PkNRT3.4*. As the only member of the PkNPF3 family, the expression of *PkNPF3.1* was found to be highest in the bud, followed by the root and needle. In addition, the majority of PkNPF4 members exhibited specific expression in phloem and xylem. *PkNPF4.2* and *PkNPF4.4* showed high expression only in the xylem, while the expression of *PkNPF4.1* and *PkNPF4.5* was detected in both the phloem and xylem, indicating their crucial roles in the internal NO_3_^−^ transport in xylem and phloem loading or unloading. The expression analysis of *PkNRTs*, which ranked among the top 12 in each tissue, revealed that *PkNPF5.2*, *PkNPF5.21*, *PkNPF6.1*, and *PkNPF8.5* showed high expression in at least five tissues (Figure 5B), suggesting potential roles in governing the fundamental function of N element transport in Korean pine. In contrast, the expression levels of nine genes (*PkNPF1.4*, *PkNPF1.6*, *PkNPF4.6*, *PkNPF5.24*, *PkNPF6.3*, *PkNPF8.2*, *PkNPF8.3*, *PkNRT2.1*, and *PkNRT2.3*) were consistently low in each tissue (Figure 5C). These findings suggest potential roles of *PkNRTs* in the absorption and transport of NO_3_^−^ within specific tissues, thereby facilitating N utilization during the growth of Korean pine.

### 2.7. Effects of N Treatment on Growth of Korean Pine Seedlings

**Figure 6:**
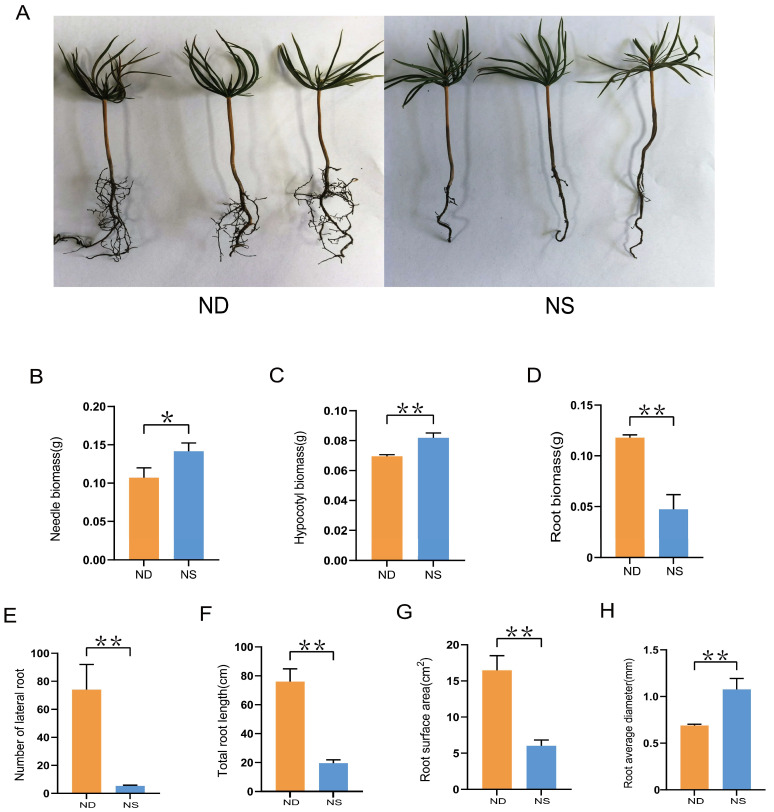
Effect of nitrogen treatment on the growth of Korean pine seedlings. (**A**) Phenotype of seedlings treated with or without nitrogen for 12 weeks. (**B**–**D**) Biomass of needle, hypocotyl, and root under ND and NS conditions. (**E**) Number of lateral roots. (**F**) Total root length of seedlings. (**G**) Root surface area. (**H**) Root mean diameter. Bars show means of three biological replicates ± standard error. Asterisk indicates significant difference between nitrogen treatment using Student’s *t*-test (* *p* < 0.05; ** *p* < 0.01).

To investigate the impact of N on the growth of Korean pine seedlings, 1-month-old seedlings were treated with either a N-containing Hoagland nutrient solution (referred to as ‘NS’ below) or a N-deficient solution (referred to as ‘ND’ below) for 12 weeks. No morphological changes were visible in the needle and hypocotyl of seedlings in response to different N treatments, whereas the lateral root growth of the seedlings under ND treatment exhibited a significant increase (Figure 6A). The biomass of the seedlings was further evaluated under ND and NS treatments. The biomass of the needle and hypocotyl significantly increased under the NS condition, whereas the biomass of the root exhibited a significant increase under the ND condition (Figure 6B–D). The phenotypes of the seedling roots were measured to confirm the impact of N on the formation of lateral roots. The number of lateral roots, total root length, and root surface area of the seedlings were significantly increased under the ND condition (Figure 6E–G). However, the root diameter of the seedlings decreased upon nitrogen deprivation (Figure 6H).

### 2.8. Expression Profiles of PkNRTs Under Different N Conditions

**Figure 7:**
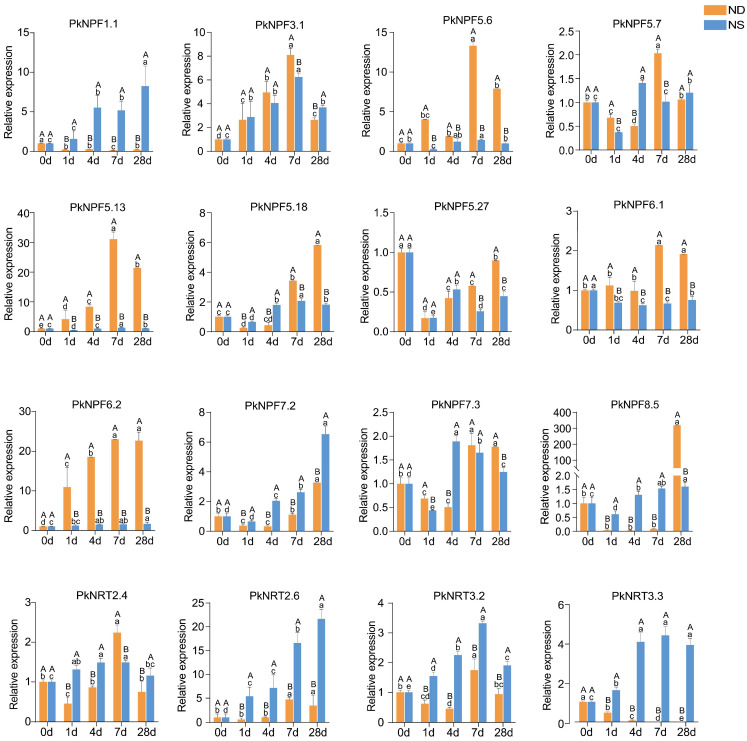
Relative expression of 16 selected *PkNRTs* under different nitrogen conditions. The expression levels of selected *PkNRTs* in roots of Korean pine seedlings after 1 d, 4 d, 7 d, and 28 d under NS and ND treatment was calculated by qRT-PCR, with *PkActin* as the reference gene. Capital letters indicate significant differences in expression levels between the two different treatments (ND and NS) at the same time (*p* < 0.05), while lowercase letters denote significant differences in expression levels at different times (0 d, 1 d, 4 d, 7 d, and 28 d) under the same treatments (*p* < 0.05). Bars show the means of three biological replicates ± standard error.

To investigate the roles of *PkNRTs* under N treatment, we subjected seedlings to a 28-day N treatment to elucidate the time-dependent expression patterns of root-specific expressed *PkNRTs* (Figure 7). The following genes showed increased expression in response to NS treatment: *PkNPF1.1*, *PkNPF3.1*, *PkNPF5.18*, *PkNPF7.2*, *PkNPF7.3*, *PkNRT2.4*, *PkNRT2.6*, *PkNRT3.2*, and *PkNRT3.3*. Meanwhile, the expression levels of *PkNPF5.27* and *PKNPF6.1* were observed to decrease. Conversely, some genes were induced under the ND condition (e.g., *PkNPF3.1*, *PkNPF5.6*, *PkNPF5.13*, *PkNPF5.18*, *PKNPF6.1*, *PkNPF6.2*, *PkNPF8.5*, *PkNRT2.4*, *PkNRT2.6*, and *PkNRT3.2*), whereas reduced expression was observed for *PkNPF1.1*, *PkNPF5.27*, and *PKNRT3.3*. Based on a qRT-PCR analysis, we observed the induction of *PkNPF3.1*, *PkNPF5.18*, *PkNPF7.2*, *PkNPF7.3*, *PkNRT2.6*, and *PkNRT3.2* through both ND and NS treatment during root development. In addition, the expression levels of *PkNPF5.6*, *PkNPF5.13*, *PkNPF6.1*, and *PkNPF6.2* were sharply upregulated in response to ND treatment, whereas the expression levels of *PkNPF1.1*, *PkNRT2.6*, and *PkNRT3.3* were robustly induced in response to N supply and higher than those observed under the ND condition. Notably, the expression of *PkNPF6.1* was induced by N starvation and repressed by N supply, whereas *PkNPF1.1* and *PkNRT3.3* exhibited the opposite expression pattern during the prolonged N treatment. The expression patterns of *PkNRTs* varied under ND and NS treatment, implying the potential diverse roles of *PkNRTs* in the process of lateral root development.

## 3. Discussion

N is widely used not only in the cultivation of agricultural crops, but also in plantation silviculture. Forest growth in northern China is currently limited by drought and low N availability [28]. Korean pine plays a dominant role in the formation of broad-leaved Korean pine mixed forest (BKF), which is a typical type of forest vegetation found in the northeast of Asia [29]. The response of Korean pine growth to climate change, specifically in relation to continuous N deposition and drought, has been extensively investigated [30,31]. However, the molecular mechanism underlying N absorption and transportation in Korean pine remains poorly understood. The majority of plants acquire N through their roots, with NO_3_^−^ serving as the primary N source in soil. The NRT proteins have been identified as crucial NO_3_^−^ transporters involved in the uptake and translocation of NO_3_^−^ in plants. Here, we performed the first comprehensive characterization of the NRT gene family and provided novel insights into its functional roles under different N conditions.

In higher plants, the members of the NRT family are abundantly present throughout the genome, with the majority belonging to the NRT1/NPF subfamily and exhibiting a high sequence similarity [32,33,34]. Our genome-wide analysis revealed a total of 76 *PkNRT* genes in Korean pine, with the majority (66/76) belonging to the NPF/NRT1 subfamily (Figure 1). The members of the NPF subfamily were further classified into eight clusters based on distinct structural domains identified in Ginkgo [35]. According to the phylogenetic analysis, the NRT proteins in Korean pine exhibited a closer clustering with those in Ginkgo, while *PkNRTs* exhibited a more distant evolutionary relationship with three model plants in angiosperms in the collinearity analysis (Figure 4 and Figure S1). The homology of *NRT* genes between Ginkgo and Poplar is closer than that between Ginkgo and Arabidopsis [35]. The findings indicate that there may be functional diversity within the NRT family in gymnosperms, as evidenced by significant differences in evolutionary trajectories between gymnosperms and angiosperms.

The expansion of gene families invariably leads to the tandem duplication of gene clusters within the genome. Variations in the number of PkNPF subfamily members were observed. For instance, the PkNPF5 clade comprises 32 members, indicating a twofold increase compared to Arabidopsis [36]. Conversely, the PkNPF2 and PkNPF3 clades consist of only three members and one member, respectively, which is significantly fewer than in Arabidopsis and other plants [16,37,38]. Additionally, high expression levels of NPF5 subfamily members across a wide range of tissues suggest that the NPF5 family plays multiple roles in nitrogen uptake and transport. Additionally, the functional diversification of the NPF5 family in Korean pine was complemented by the expansion and diversification of the NPF2 and NPF3 families in Arabidopsis during evolution. This evolutionary process resulted in a relative reduction in the number of NPF5 members and an expansion of the NPF2 and NPF3 families in Arabidopsis. This suggests that gene expansion and deletion events have also occurred within the PkNPF subfamily. Similar findings were also observed in other plants, such as Ginkgo [35], Pineapple [9], wild soybean [39], and radish [16]. Moreover, we found that most *PkNRTs* exhibited a tandem distribution pattern across the chromosomes, including members in NPF1, NPF2, NPF5, NPF8, NRT2, and NRT3 (Figure 3). The members involved in tandem regions exhibited a more closely related evolutionary relationship in the phylogenetic analysis, indicating potential functional redundancy among these genes.

Given that all members of the NRT gene family are transmembrane proteins, the tissue-specific expression of *NRTs* provides a comprehensive understanding of their functional roles across diverse tissues and developmental stages [21]. Here, we elucidated the gene expression patterns of *PkNRTs* across different tissues. The majority of *PkNRTs* exhibited high expression levels in needles or roots, indicating their crucial role in NO_3_^−^ uptake by the roots and subsequent redistribution to the needles for various physiological processes in plants (Figure 5). Notably, the expression of members within the NPF4 clade predominantly occurred in the xylem and phloem, whereas the NPF2 clade exhibited high expression in both the needle and phloem, indicating that these two clades of *PkNPFs* function in NO_3_^−^ transport in aboveground tissues [40]. Furthermore, the expression of several of these genes is significantly higher in almost all tissues, indicating their potential fundamental roles in NO_3_^−^ uptake and transport. In contrast, nine *PkNRTs* showed low expression levels in all detected tissues. Some of these genes, such as *PkNPF4.6*, *PkNPF6.3*, and *PkNPF8.2* possess fewer *cis*-regulatory elements in their promoters. The promoters of other genes such as *PkNPF1.4*, *PkNPF1.6*, and *PkNPF8.3* contain numerous MeJA-responsive elements, while promoters of *PkNPF2.1 and PkNPF2.3* harbor drought-responsive elements, indicating that these genes may play roles in plant stress responses or hormonal regulation. However, it should be noted that the expression of *NRTs* may be regulated by many other factors, such as soil pH, drought, cold, and salinity, as well as hormones [41,42,43,44]. In fact, we have identified numerous hormone-, drought-, and cold-responsive *CREs* in the promoter region of *PkNRTs* (Figure S2). Therefore, the lower expression of *PkNRTs* may also indicate their function in other undetected tissues, such as the callus or bark, or in response to hormones or cold. Further investigations will be conducted to elucidate the expression pattern of *PkNRTs* in response to abiotic stress and hormone stimuli. Although further functional analysis is required to identify the roles of *PkNRTs*, their expression patterns may provide insight into their potential functions in NO_3_^−^ acquisition and transport within Korean pine.

The presence of NO_3_^−^ is crucial for root growth, development, and architecture, especially in relation to lateral root development [45]. A high and consistent concentration of NO_3_^−^ in the growth medium inhibits the development of lateral roots, which is associated with the accumulation of NO_3_^−^ and N metabolites within the plants [46]. In many plants, the stimulation of lateral root growth was observed under low NO_3_^−^ conditions, with a significant increase in lateral root number, total root length, and root biomass [47,48,49]. Similarly, we observed that N deficiency promoted lateral root growth in Korean pine seedlings, as evidenced by an increase in their number and biomass (Figure 6). Expression changes in response to different N concentrations reveal the potential roles of NRT proteins for NO_3_^−^ absorption and transport. The expression of high-affinity NO_3_^−^ transporters, such as *NRT2.1*, *NRT2.4*, *NRT2.5*, and *NPF6.3*, was induced in response to N starvation to enhance root NO_3_^−^ acquisition [11,18]. The majority of members in the NPF/NRT1 subfamily, such as *NPF7.3*, *NPF4.6*, and *NPF2.7*, which function as low-affinity NO_3_^−^ transporters, exhibited an increased expression in response to N supply [50,51]. In the present work, the expression of 16 *PkNRTs*, which have been identified to exhibit high or specific expression in the root, was further detected under the ND and NS conditions. The expression levels of *PkNRTs* in seedling roots displayed differential patterns (Figure 7). Among the *PkNRTs*, the significant upregulation of *PkNPF1.1*, *PkNRT2.6*, and *PkNRT3.3* expression under NS conditions suggested their critical roles as low-affinity NO_3_^−^ transporters in root NO_3_^−^ uptake. Meanwhile, *PkNPF5.6*, *PkNPF5.13*, *PkNPF6.1*, and *PkNPF6.2* were identified as potential members of HATS for efficient NO_3_^−^ uptake due to their robustly upregulated expression levels in response to ND treatment, which exhibited a strong correlation with lateral root development.

## 4. Materials and Methods

### 4.1. Identification of NRT Gene Family in Korean Pine

In order to identify all NRT gene family members in Korean pine, a BLASTP search was conducted in the full protein sequence isolated from *P. koraiensis* genome by using the NRT protein sequences of *Arabidopsis thaliana*, *Ginkgo biloba*, and *Populus trichocarpa* as a query retrieved from the TAIR (https://www.arabidopsis.org/, accessed on 17 July 2024), Ginkgo DB (https://ginkgo.zju.edu.cn/genome/ftp/, accessed on 17 July 2024), and phytozome databases (https://phytozome-next.jgi.doe.gov/, accessed on 17 July 2024), respectively. The complete genome of *P. koraiensis* and annotation information were obtained from our group. The protein sequences of *P. koraiensis* utilized in this study can be found in Table S8. The sequences were submitted to the Pfam database (http://pfam.xfam.org/, accessed on 18 July 2024) for screening and identification of the PTR2 (PF00854) core domain in the NPF subfamily, the MSF_1 (PF07690) core domain in the NRT2 subfamily, and the NAR2 (PF16974) core domain in the NRT3 subfamily. After eliminating the redundant sequences, the biophysical properties, such as amino acid length, theoretical isoelectric point (pI), molecular weight (Mw), and grand average hydropathy (GRAVY) for each *PkNRT* was calculated using the ProtParam tool available on the ExPASy Server (https://web.expasy.org/protparam/, accessed on 18 July 2024). The TMHMM-2.0 tool (https://services.healthtech.dtu.dk/service.php?TMHMM-2.0, accessed on 19 July 2024) was employed to predict the transmembrane regions of the putative *PkNRTs*.

### 4.2. Phylogenetic Analysis and Structural Characterization of PkNRTs

To investigate the phylogenetic relationships of the *PkNRTs*, multiple sequence alignment of the NRT protein sequences from *A. thaliana*, *P. trichocarpa*, *G. biloba*, and *P. koraiensis* was carried out using ClustalW. The phylogenetic tree was constructed using the neighbor-joining method with 1000 bootstrap replicates in MEGA 11.0 software. The integrity of the conservative domains of the *PkNRTs* was verified and analyzed using the batch CD-search tool at the National Center for Biotechnology Information (NCBI) (https://www.ncbi.nlm.nih.gov/Structure/bwrpsb/bwrpsb.cgi, accessed on 19 July 2024). MEME Version 5.5.0 (https://meme-suite.org/meme/tools/meme, accessed on 19 July 2024) was utilized for conservative motif analysis, with a maximum cardinal number of 10 set for NPF and NRT2, and 4 set for NRT3. The detailed sequence information of these motifs is presented in Table S1. The General Feature Format (GFF) annotation files were used for gene structure analysis. TBtools visualizes *PkNRT* gene structures, conservative motifs, and domains [52].

### 4.3. Colinearity Analysis and Calculation of Ka/Ks Values of PkNRTs

To study the collinearity relationship of the *PkNRTs*, the chromosomal locations of these candidate *PkNRTs* were obtained from the GFF annotation files, and they were generated using the TBtools 2.1 software. Gene repeat events and collinearity relationships were analyzed using the Multiple Collinear Scan Toolkit (MCScanX 1.0) [53]. The O. sativa genome was downloaded from the Rice Genome DB (https://rice.uga.edu/, accessed on 21 July 2024). The Ka/Ks calculator program in TBtools was utilized to compute the non-synonymous substitution rate (Ka), synonymous substitution rate (Ks), and Ka/Ks ratios among collinear gene pairs and tandemly duplicated gene pairs.

### 4.4. Analysis of CREs in the Promoters of PkNRTs

The 2000 bp promoter region, located upstream of the transcription start site (TSS) of *PkNRTs*, was loaded into the PlantCARE (http://bioinformatics.psb.ugent.be/webtools/plantcare/html/, accessed on 22 July 2024) database to identify potential cis-regulatory elements (*CREs*).

### 4.5. Transcriptome Sequencing for Tissue-Specific Abundance Analysis

Current-year needles, buds, shoots, phloem, xylem, and roots were collected from a 200-year-old Korean pine. The phloem and xylem were obtained from the primary branches of the tree, while root samples were partially extracted from the soil at depths ranging from 10 to 20 cm. Each part’s samples comprised three biological replicates. All collected materials were frozen in liquid nitrogen and subsequently sent to BGI (Shenzhen, China) for transcriptome sequencing. FPKM (fragments per kilobase of exon per million reads mapped) values were calculated based on the RNA-seq data, and the average FPKM of the three replicates in each tissue was calculated. Heat maps were generated using TBtools.

### 4.6. Plant Materials and Treatments

The Korean pine seeds were obtained from the Hongwei Seed Orchard of the Lushuihe Forestry Bureau, Jilin Province, China, and their dormancy was released using the variable temperature stratification method [54]. The seeds underwent variable temperature stratification treatment (20 °C, 60% humidity for 60 days; 5 °C, 60% humidity for 90 days) to break seed dormancy. Then, they were sown in 3 × 7 pots filled with sterilized sand and placed in the flower greenhouse at 25 °C ± 2 under a 16 h photoperiod. During one month of seed germination, distilled water was used for irrigation. Then, the seedlings were subjected to a 12-week treatment with Hoagland nutrient solution (with 10 mM NO_3_^−^ + 1 mM NH_4_^+^) and a N-deficient solution (with 0 mM NO_3_^−^ and NH_4_^+^). The seedlings were watered with two solutions every 5 days. The needles, stems, and roots were collected at 12 weeks for phenotypic and physiological assays. The roots were harvested for RNA isolation at 1 day, 4 days, 7 days, and 28 days. All samples were collected in liquid N and stored at −80 °C until used.

### 4.7. Seedling Biomass and Root Morphology

The biomass of needle, hypocotyl, and root was determined by measuring their weight after being thoroughly dried at 65 °C until a constant weight was achieved. The roots were scanned with an Epson Expression 10000XL color scanner (SeikoEpson Corporation, Nagano-ken, Japan). The morphological traits of the roots were assessed through an analysis of 10 seedlings per treatment. Total length, lateral root number, root surface area, and mean diameter were determined using the rootsystem analyzer software WinRHIZO Pro 2016 (Regent Instruments Inc., Montreal, Québec, QC, Canada) [55].

### 4.8. Quantitative Real-Time PCR Analysis

According to the manufacturer’s protocol, the total RNA was extracted from the roots of Korean pine seedlings in response to N treatments using a TransZol Up Reagent (TransGen Biotech, Beijing, China). The purity and integrity of the isolated total RNA were assessed using agarose gel electrophoresis and a Nanodrop 2000 spectrophotometer (Thermo Scientific, Wilmington, NC, USA). The samples were then stored at −80 °C until further analysis. For reverse transcription, removal of genomic DNA and first-strand cDNA synthesis using oligo (dT) were carried out using a TransScript One-Step gDNA Removal and cDNA Synthesis SuperMix kit (TransGen Biotech, Beijing, China). Quantitative real-time PCR (qRT-PCR) was performed with the Perfectstart Green qPCR SuperMix (TransGen Biotech, Beijing, China) by using a LightCycler^®^ 480 System (Roche, Basel, Switzerland). The relative expression level was determined through the 2^−∆∆CT^ method [56] with PkActin used as the internal control. All primers used for qRT-PCR analysis are listed in Table S7. Three biological and three technical replicates were performed for each analysis.

### 4.9. Statistical Analysis

Statistical analysis was carried out through a two-way analysis of variance (ANOVA) at the *p* < 0.05 level of significance using SPSS 27.0 software (SPSS Inc., Chicago, IL, USA). The values were determined using the mean ± standard deviation (SD) from three biological replicates. Figures were generated using Graphpad Prism 9.

## Figures and Tables

**Table 1:** Characteristics of the *PkNRT* genes in Korean pine.

Gene Name	Gene ID	No. of AA	MW (kDa)	pI	GRAVY	No. of TMs
*PkNPF1.1*	*Pkor04G00574*	633	69.73	8.69	0.142	10
*PkNPF1.2*	*Pkor04G00573*	630	69.28	8.92	0.141	9
*PkNPF1.3*	*Pkor04G00568*	635	70.17	9.34	0.17	10
*PkNPF1.4*	*Pkor04G04403*	639	70.13	9.25	0.148	10
*PkNPF1.5*	*Pkor04G00571*	639	70.45	9.35	0.096	8
*PkNPF1.6*	*Pkor04G00570*	639	70.33	9.21	0.088	8
*PkNPF1.7*	*Pkor04G00569*	639	70.33	9.21	0.088	8
*PkNPF2.1*	*Pkor10G02120*	661	73.07	9.21	0.201	10
*PkNPF2.2*	*Pkor10G02119*	638	70.83	8.53	0.125	8
*PkNPF2.3*	*Pkor10G02121*	658	72.67	7.12	0.159	10
*PkNPF3.1*	*Pkor12G01674*	648	71.75	9.17	0.154	11
*PkNPF4.1*	*Pkor01G02841*	564	63.08	8.82	0.36	11
*PkNPF4.2*	*Pkor01G03784*	574	64.51	8.29	0.35	12
*PkNPF4.3*	*Pkor11G00219*	580	64.53	8.46	0.3	11
*PkNPF4.4*	*Pkor08G03209*	623	69.08	7.61	0.234	12
*PkNPF4.5*	*Pkor08G02109*	607	66.91	9.02	0.171	12
*PkNPF4.6*	*Pkor08G03210*	606	67.09	9.01	0.144	11
*PkNPF5.1*	*Pkor10G00974*	618	69.15	9.1	0.255	10
*PkNPF5.2*	*Pkor08G01587*	608	67.49	9.27	0.179	9
*PkNPF5.3*	*Pkor08G01589*	598	66.37	9.14	0.228	9
*PkNPF5.4*	*Pkor02G03103*	609	68.36	9.15	0.214	11
*PkNPF5.5*	*Pkor02G03104*	537	59.25	9.47	0.277	10
*PkNPF5.6*	*Pkor02G01331*	629	70.39	9.27	0.163	12
*PkNPF5.7*	*Pkor02G01336*	623	69.98	9.09	0.173	10
*PkNPF5.8*	*Pkor02G03105*	623	69.81	9.33	0.232	10
*PkNPF5.9*	*Pkor02G01335*	622	69.88	9.35	0.2	10
*PkNPF5.10*	*Pkor05G02326*	614	68.27	9.05	0.269	10
*PkNPF5.11*	*Pkor06G02154*	614	68.49	9.02	0.264	9
*PkNPF5.12*	*Pkor06G00882*	605	67.07	9.14	0.301	12
*PkNPF5.13*	*Pkor06G00883*	607	67.27	8.54	0.382	12
*PkNPF5.14*	*Pkor09G01222*	612	68.18	8.95	0.29	11
*PkNPF5.15*	*Pkor06G00884*	606	67.37	8.89	0.323	12
*PkNPF5.16*	*Pkor06G03064*	611	68.28	7.54	0.296	12
*PkNPF5.17*	*Pkor06G00881*	607	68.42	9.05	0.254	12
*PkNPF5.18*	*Pkor06G03063*	608	67.87	8.88	0.305	12
*PkNPF5.19*	*Pkor06G03062*	608	67.78	8.59	0.318	12
*PkNPF5.20*	*Pkor12G00815*	559	61.88	5.83	0.393	10
*PkNPF5.21*	*Pkor12G01382*	605	67.84	5.92	0.248	12
*PkNPF5.22*	*Pkor07G00455*	578	64.67	5.86	0.274	9
*PkNPF5.23*	*Pkor07G00456*	579	64.83	6.32	0.364	10
*PkNPF5.24*	*Pkor07G02566*	547	61.45	5.71	0.277	8
*PkNPF5.25*	*Pkor07G02567*	606	68.19	8.79	0.227	9
*PkNPF5.26*	*Pkor07G00457*	590	66.15	8.82	0.253	9
*PkNPF5.27*	*Pkor07G00460*	592	66.1	8.12	0.338	10
*PkNPF5.28*	*Pkor07G00458*	595	66.58	7.53	0.33	10
*PkNPF5.29*	*Pkor07G00459*	595	66.58	6.86	0.335	10
*PkNPF5.30*	*Pkor07G00461*	578	64.18	7.91	0.304	10
*PkNPF5.31*	*Pkor07G02568*	576	63.74	8.44	0.325	10
*PkNPF5.32*	*Pkor07G00462*	576	63.83	8.27	0.335	10
*PkNPF6.1*	*Pkor12G01587*	610	66.98	8.43	0.243	11
*PkNPF6.2*	*Pkor11G00350*	601	66.65	8.96	0.242	11
*PkNPF6.3*	*Pkor11G02733*	597	66.53	8.28	0.209	11
*PkNPF7.1*	*Pkor11G02681*	598	65.67	7.96	0.301	12
*PkNPF7.2*	*Pkor11G00148*	626	69.15	8.52	0.208	12
*PkNPF7.3*	*Pkor09G02450*	630	70.58	8.2	0.082	11
*PkNPF7.4*	*Pkor09G02643*	622	69.64	6.07	0.134	12
*PkNPF8.1*	*Pkor09G00733*	589	65.58	8.97	0.111	9
*PkNPF8.2*	*Pkor09G00732*	571	63.61	8.22	0.136	10
*PkNPF8.3*	*Pkor05G01620*	596	66.25	5.48	0.212	10
*PkNPF8.4*	*Pkor05G01614*	596	66.17	5.25	0.237	10
*PkNPF8.5*	*Pkor05G01606*	594	65.59	6.28	0.204	11
*PkNPF8.6*	*Pkor05G01605*	594	65.97	6.15	0.258	11
*PkNPF8.7*	*Pkor01G04179*	566	63.29	8.23	0.258	10
*PkNPF8.8*	*Pkor09G00734*	616	68.79	8.53	0.069	10
*PkNPF8.9*	*Pkor09G00735*	589	66.21	8.86	0.128	9
*PkNPF8.10*	*Pkor09G00737*	589	66.15	8.74	0.117	10
*PkNRT2.1*	*Pkor08G00516*	533	58	8.69	0.3	10
*PkNRT2.2*	*Pkor12G01755*	533	58	8.69	0.3	10
*PkNRT2.3*	*Pkor12G02554*	533	58	8.69	0.3	10
*PkNRT2.4*	*Pkor12G02553*	533	57.87	8.39	0.297	10
*PkNRT2.5*	*Pkor12G01756*	533	57.95	8.14	0.333	10
*PkNRT2.6*	*Pkor09G01906*	548	58.63	8.17	0.353	10
*PkNRT3.1*	*Pkor09G03181*	268	29.26	9.18	0.018	1
*PkNRT3.2*	*Pkor09G01715*	207	22.58	9.73	0.173	1
*PkNRT3.3*	*Pkor09G01713*	214	23.98	9.25	0.012	1
*PkNRT3.4*	*Pkor09G01714*	214	23.49	9.64	−0.037	1

Note: No. of AAs represents the number of the amino acids in each protein; molecular weight, Mw; isoelectric point, pI. GRAVY represents grand average hydropathy. No. of TMs, transmembrane domains.

## Data Availability

The data that support the findings of this study are available in the Supplementary Materials of this article.

## Supplementary Materials

The following supporting information can be downloaded at https://www.mdpi.com/article/10.3390/plants14020238/s1: Figure S1: Collinearity analysis of *NRTs* between Korean pine, rice, and Arabidopsis; Figure S2: The distribution and number of cis-regulatory elements (*CREs*) in the PkNRT gene promoter; Table S1: Detailed information about motif sequences; Table S2: Gene structure information of *PkNRTs*; Table S3: Selective pressure on gene pairs between Korean pine, Poplar, and Ginkgo; Table S4: Selective pressure on the tandem duplicated gene pairs in *PkNRTs*; Table S5: The potential *cis*-regulatory elements in the promoter region of 76 *PkNRTs*; Table S6: Expression levels of *PkNRTs* in different tissues; Table S7: List of qRT-PCR primers in selected *PkNRTs*; Table S8: The *NRT* genes of *Pinus koraiensis.*
