# Supplementary material for: Genome-Wide Identification and Expression Analysis of PkNRT Gene Family in Korean Pine (Pinus koraiensis)
Source: Plants (Basel). 2025 Jan 16;14(2):238. doi: 10.3390/plants14020238 (PMC11769194; doi:10.3390/plants14020238)
Supplement: Supplementary file 1 [file plants-14-00238-s001.zip › Supplementary figure.pdf]

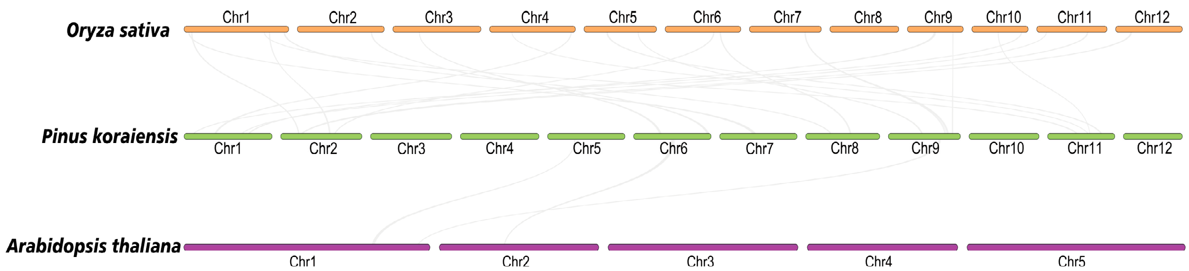

Figure S1. Co-linearity analysis of NRTs between Korean pine, rice and Arabidopsis. The gray lines in the background represent collinear gene pairs observed between Korean pine and the other two species.

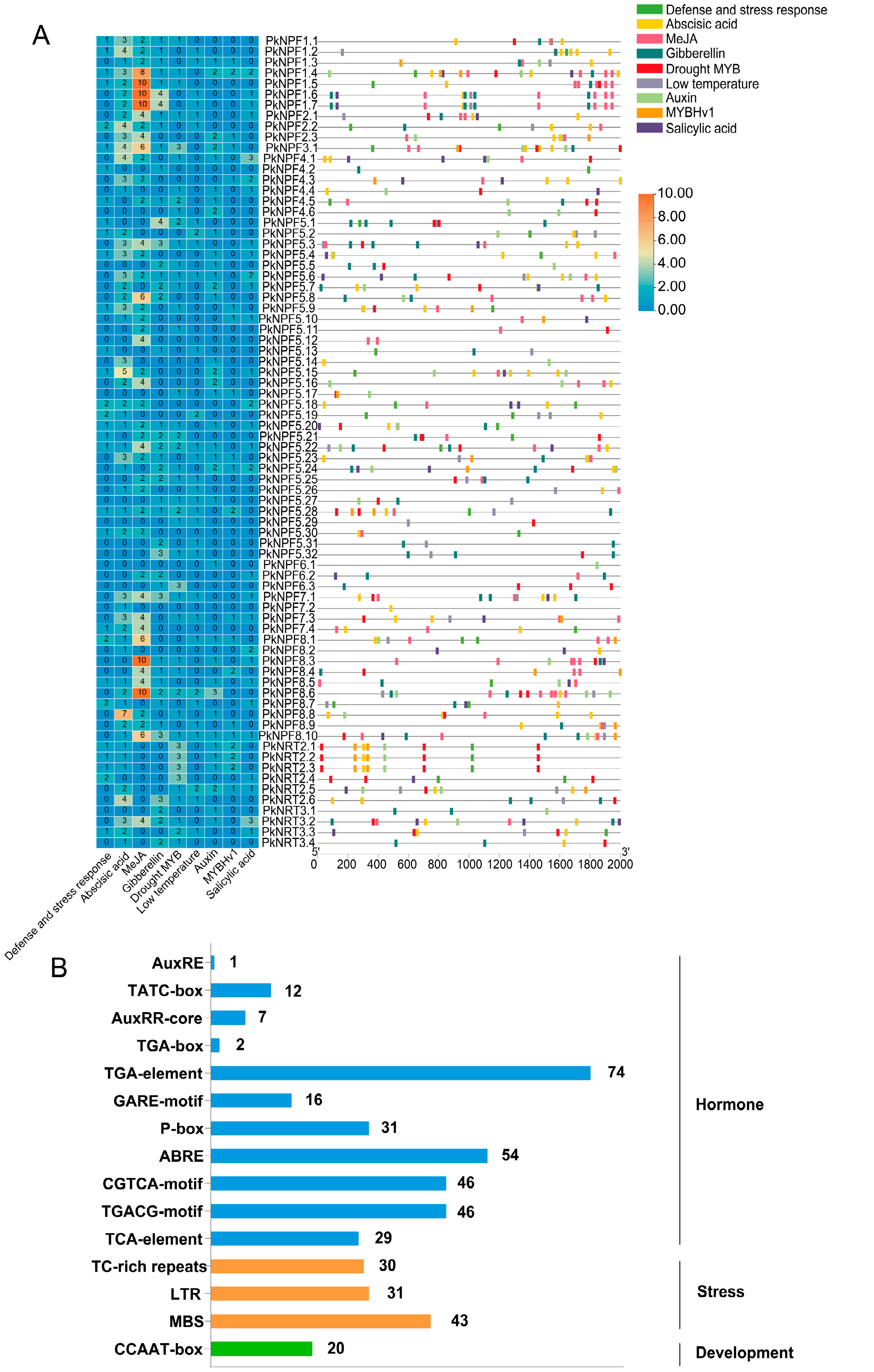

Figure S2. The distribution and number of *cis*-regulatory elements (CREs) in PkNRT gene promoter. (A) The heat map on the left contained CREs occurrence frequency on the promoter of *PkNRT* genes. The number represented the element frequency of occurrence. The distribution of CREs in PkNRT promoter was shown on the right.

Different colored columns on the lines represented different types of CREs. (B) Total number of each identified CRE in the promoter of *PkNRT* genes.
